# Characterizing the credentialed bachelor's-level behavioral health support workforce: a national descriptive study

**DOI:** 10.1093/haschl/qxag222

**Published:** 2026-09-11

**Authors:** Brianna M Lombardi, Lisa D Zerden, Maria Gaiser

**Affiliations:** Department of Family Medicine, University of North Carolina at Chapel Hill, Chapel Hill, NC 27599, United States; School of Social Work, University of North Carolina at Chapel Hill, Chapel Hill, NC 27599, United States; Cecil G. Sheps Center for Health Services Research, University of North Carolina at Chapel Hill, Chapel Hill, NC 27599, United States; School of Social Work, University of North Carolina at Chapel Hill, Chapel Hill, NC 27599, United States; Cecil G. Sheps Center for Health Services Research, University of North Carolina at Chapel Hill, Chapel Hill, NC 27599, United States; Cecil G. Sheps Center for Health Services Research, University of North Carolina at Chapel Hill, Chapel Hill, NC 27599, United States

**Keywords:** behavioral health workforce, state policy, workforce development, licensure and development

## Abstract

**Introduction:**

Persistent shortages of the behavioral health workforce have prompted interest in expanding roles for bachelor's-level behavioral health support workers as a strategy for increasing care. States vary in how they regulate this workforce. This study characterized how US states define the bachelor's-level behavioral health workforce through credentialing structures, educational requirements, and scope of practice.

**Methods:**

Data were systematically abstracted from credentialing authorities, state administrative codes, state Medicaid programs, and government webpages using a standardized extraction guide.

**Results:**

Twenty-nine states had at least one bachelor's-level credential. A total of 50 credentials met inclusion criteria and were grouped into 2 categories: 20 licensed or certified credentials (9 states); 30 roles were defined solely in state administrative codes or other state guidance (22 states). Educational requirements differed across states, with most roles specifying degrees in human services or related fields and required practice hours ranging from zero to 10 000 hours of supervised experience. Nearly half of credentialed roles permitted multiple entry pathways, allowing work experience to substitute for formal education.

**Conclusion:**

Findings reveal substantial variation in how states define and regulate this workforce, creating barriers to workforce planning, interstate mobility, and service delivery consistency.

The United States (US) faces a well-documented shortage of licensed graduate-level behavioral health clinicians that constrains access to mental health and substance use services across care settings.^1,2^ Rates of behavioral health treatment remain stagnant as demand for mental health services continues to rise, with only half of individuals that have a mental health need receiving treatment and many forgoing care due to wait times and costs.^3^ In response, states have accelerated efforts to expand behavioral health workforce capacity through a range of strategies that include new education and career pathways, regulatory reform, and investments in workforce development and incentive programs.^4-6^

One emerging strategy that states are using to increase behavioral health service capacity is expanding the size of the behavioral health workforce through formalization of bachelor's-level behavioral health support roles.^5,6^ This strategy recognizes that the behavioral health workforce is disproportionately concentrated at the graduate level and lacks the layered workforce structure common in other health professions, whereby a substantial mid-level workforce supports care delivery at scale.^7^ Prior work supports the use of task-sharing models in which individuals without advanced clinical training are equipped to deliver structured psychosocial interventions under supervision,^8^ and in most states individuals with bachelor's-level training already fill critical roles in direct in-home behavioral health care, inpatient psychiatric settings, and mobile crisis services.^6,7^ However, the integration of this bachelor's-level workforce and the roles these individuals fill in the behavioral health system vary widely across states, shaped by differences in regulatory authority, licensure/credentialing, and payment coverage. Credentialing, a formal process that uses an established series of guidelines to verify that an individual has achieved a baseline level of competency and is authorized to carry out their duties, and is intended to promote consistency in qualifications, training, and practice standards across jurisdictions.^9^ Despite the growing recognition of the bachelor's-level behavioral health workforce as a potential lever for building capacity and expanding access to care, not all states formally credential, regulate, or define these roles.

States have approached integrating bachelor's-level roles into the workforce in different ways. For example, Washington enacted legislation to establish a formal bachelor's-level Behavioral Health Support Specialist credential that includes an educational/training pathway, a regulated certificate, and inclusion into the Medicaid state plan.^8,10^ Utah created a licensed Behavioral Health Coach credential for individuals with a related 4-year degree,^11^ and states like Massachusetts have incorporated bachelor's-level training for direct behavioral health roles without formalizing a state-regulated credential.^12^ Yet these examples remain exceptions, and no systematic description of how states define, credential, and regulate such roles currently exists. This lack of uniformity across states in titles, educational requirements, training expectations, and regulatory oversight closely parallels challenges that have been documented in the addiction workforce. Research examining the high-school- and bachelor's-level addiction counselor workforce identified 160 distinct credentials across all 50 states and the District of Columbia, with wide variability in minimum educational requirements, training hours, supervision standards, and Medicaid reimbursement eligibility.^13^ This variability is consequential because credentialing directly shapes who enters the behavioral health workforce, how they are trained, what they are authorized to do, and how they advance professionally.^13-15^ Social work, however, has developed a more unified structure for bachelor of social work (BSW) licensure. Of the 42 states that have BSW licensure, all require completion of a BSW from a Council on Social Work Education-accredited program and passing a standardized licensing exam.^16^

While credentialing structures for addiction counselors and licensed BSWs have received attention, far less is known about how states credential and regulate the growing bachelor's-level behavioral health support role. Currently, no comprehensive description of how states credential or regulate the bachelor's-level behavioral health support role exists, making it difficult to discern how these workers are filling states' workforce needs or where policy gaps remain. As such, this study sought to characterize how states define the bachelor's-level behavioral health support role workforce through credentialing structures, educational requirements, scope of practice/authorized services, and regulatory authority. Findings highlight legislative trends and policy strategies that illustrate how states can adopt or expand bachelor's-level workforce credentials to address behavioral health capacity challenges.

## Methods

We conducted a 50-state comparative analysis of credentialing requirements for the bachelor's-level behavioral health support role workforce to understand (1) how states define bachelor's-level behavioral health support roles, including through licensure, certification, administrative code, and other state policy; (2) the credentialing or regulatory authority responsible for oversight and regulation of those roles; (3) the education, training, and experience requirements associated with each credential; and (4) the scope of practice or authorized services permitted under each credential.

### Definitions and categorization of bachelor's-level workforce

For the purposes of this analysis, a credential is defined as a formal qualification required to serve in a specific bachelor's-level behavioral health support role, including licensure, certification, or eligibility criteria established through a state's administrative code.^9^ A credentialing authority refers to the body responsible for establishing and overseeing those requirements, which may include occupational licensing boards, state agency licensing boards, or state affiliates of national credentialing organizations. States can formally recognize bachelor's-level behavioral health support roles through 4 primary mechanisms: (1) *licensure*, administered through a state occupational or agency licensing board, which typically defines a specific scope of practice that delineates the services a worker is authorized to perform and those they are not authorized to perform; (2) *certification*, offered through a state credentialing authority or affiliate of a national credentialing organization, which verifies that an individual has met a defined set of educational and training standards but does not always carry a formally defined scope of practice; (3) *administrative code*, in which a state legislature or agency establishes definitions, titles, and requirements for these roles through rulemaking without a corresponding licensure or certification structure; or (4) *state agency policy or program documents*, including state agency operational standards, Medicaid provider manuals, and managed care contracts, through which state agencies or their contracted partners define eligible provider types and staffing qualifications outside of the formal rulemaking process.

Credentialing authorities were classified as *internal* when the oversight body is a state government agency, such as a state department of health or department of human services, and as *external* when the credentialing body is an independent organization operating outside of state government, such as a professional certification board, a state affiliate of a national credentialing organization, or a professional association.

Bachelor's-level roles were identified as having alternative entry pathways through which an individual could meet credentialing requirements through different combinations of education and experience, such as substituting accrued work experience hours for formal educational attainment.^17^ For example, some states allow individuals with an associate's degree to qualify for a bachelor's-level credential by completing additional supervised practice hours. Credentials were included when a bachelor's degree represented a standard or primary pathway to credentialing, even if states also permitted alternative pathways based on combinations of education and experience. Excluded from analysis were credentials for which minimum educational requirements were at or below the high school level (e.g., a GED or equivalent) or that required a graduate-level degree as the minimum qualification for entry. Bachelor of social work licensure was also excluded from the analysis if the only pathway to licensure was a BSW, as this credential is well formalized and documented.^16^

### Data collection

A standardized data extraction guide was developed by the project team to systematically identify, classify, and abstract key variables across states, including credential titles; credential descriptions; minimum educational degree requirements; required practice and supervision hours; scope of practice/authorized services; and the credentialing or regulatory authority. Systematic abstraction of state-specific data was completed through review of credentialing authority websites, state administrative codes, state Medicaid websites, and other government webpages. Data were extracted by 2 team members between November 2024 and May 2025 and reviewed by all authors for accuracy and consistency. Discrepancies between the 2 abstractors were resolved through discussion and consensus. When consensus could not be reached, a third senior team member participated in the discussion to facilitate resolution. The finalized abstraction was subsequently reviewed by the full study team to ensure consistency and accuracy. Additional details regarding the data extraction process are provided in Appendix A.

### Analysis

Each identified role was classified according to how it was established or recognized by the state. State-specific information, including allowable services and supervised practice requirements, were organized into standardized categories to allow for comparison of credential characteristics across states. Credentials formally established through licensure or certification were categorized separately from bachelor's-level occupations established through administrative code, Medicaid provider manuals, state agency guidance, or other documented state policies. Findings were compiled in tables and descriptively compared to document similarities and differences in credential titles, educational requirements, scope of practice, and regulatory structures across states.

## Results

Twenty-nine states had at least one bachelor's-level behavioral health credential, with 50 different roles meeting criteria for inclusion in the policy analyses. Most states only had a single credential (*n* = 19; 66%), with 10 states defining 2 or more roles. Nearly half (*n* = 21; 42%) of the identified roles offered alternative pathways to entry. In this context, having more than one pathway meant there were differing education and experiential options that could result in an individual being awarded the same credential.

### Licensed and certified bachelor's-level credentials

A total of 20 bachelor's-level licenses or certifications were identified in 9 states (AK, FL, MS, NH, OK, OR, UT, VA, and WA), of which 4 states (AK, OR, VA, and WA) offered multiple bachelor's-level credential types (see Table 1). Eighteen of these credentials were certifications, with only 2 encompassing a license (NH, UT), most of which (*n* = 12; 60%) were regulated by external boards.

**Table 1.** Licensed and certified bachelor's-level behavioral health occupations, by state (*n* = 9).

State	Title	Credential type	Credentialing body	Degree required	Practice hours	Scope of practice/Authorized services	Alternative entry pathway
AK	Behavioral Health Technician	Certification	External—Alaska Commission for Behavioral Health Certification	Related/relevant field	N/A	Treatment support under supervision (SO)	Yes
AK	Behavioral Health Counselor I	Certification	External—Alaska Commission for Behavioral Health Certification	Related/relevant field	1 year full-time	NS	Yes
AK	Behavioral Health Counselor II	Certification	External—Alaska Commission for Behavioral Health Certification	Related/relevant field	3 yrs/6 000 hrs	NS	Yes
AK	Advanced Behavioral Health Counselor	Certification	External—Alaska Commission for Behavioral Health Certification	Related/relevant field	5 yrs/10 000 hrs	NS	Yes
AK	Behavioral Health Aide I	Certification	External—State Community Health Aide Program Certification Board	BH or human services	1 000 supervised hrs	Assessment; CM; community education and prevention	No
AK	Behavioral Health Aide II	Certification	External—State Community Health Aide Program Certification Board	BH or human services	2 000 cumulative supervised hrs	Assessment; TP; PS; community prevention	No
AK	Behavioral Health Aide III	Certification	External—State Community Health Aide Program Certification Board	BH or human services	4 000 cumulative supervised hrs	Assessment; TP; PS; clinical team leadership	No
AK	Behavioral Health Practitioner	Certification	External—State Community Health Aide Program Certification Board	BH or human services	6 000 cumulative supervised hrs	Supervision of lower-level aides; clinical team leadership	No
FL	Certified Mental Health Professional	Certification	External—Florida Certification Board	Related/relevant field	2 000 hrs (Tier 1); 4 000 hrs (Tier 2)	Assessment; TP; counseling; CM (SO)	No
MS	Community Support Specialist	Certification	Internal—Department of Mental Health	BH or mental health-related field	1 yr relevant experience	CM; wraparound facilitation; IDD support coordination	No
NH	Licensed Social Work Associate (LSW-A)	License	Internal—State Board of Mental Health Practice	BH or human services	4 000 supervised hrs	Assessment; TP; treatment under clinical supervision (SO)	Yes
OK	Certified Behavioral Health Case Manager II	Certification	Internal—Department of Mental Health and Substance Abuse Services	BH or human services	3 yrs direct MH experience	CM; referral; patient advocacy	No
OR	Qualified Mental Health Associate-Registered (QMHA-R)	Certification	External—Mental Health & Addiction Certification Board	Behavioral science or equivalent	1 000 supervised hrs	NS	Yes
OR	Qualified Mental Health Associate I (QMHA-I)	Certification	External—Mental Health & Addiction Certification Board	Nonspecified bachelor's or equivalent	1 000 supervised hrs	PS; CM; behavior management	Yes
OR	Qualified Mental Health Associate II (QMHA-II)	Certification	External—Mental Health & Addiction Certification Board	Nonspecified bachelor's or equivalent	4 000 supervised hrs	PS; CM; behavior management	Yes
UT	Behavioral Health Coach	License	Internal—Division of Professional Licensing	Related/relevant field	N/A	Supervised services within SUD or MH agency (SO)	No
VA	Qualified Mental Health Professional (QMHP)	Certification	Internal—Virginia Board of Counseling, Department of Health Professions	Nonspecified bachelor's	1 500 supervised hrs; 80 hrs didactic education	Assessment; CM; crisis services; PS; collaborative services (SO)	No
VA	Qualified Mental Health Professional Trainee	Certification	Internal—Virginia Board of Counseling, Department of Health Professions	Enrolled in or completed bachelor's program	60 hrs didactic education	Assessment; CM; crisis services; PS; collaborative services (SO)	Yes
WA	Behavioral Health Support Specialist	Certification	Internal—State Department of Health	Nonspecified bachelor's	60 hrs direct client contact within education program	Brief evidence-based interventions; treatment monitoring (SO); mild to moderate conditions only	No
WA	Certified Counselor	Certification	Internal—State Department of Health	Counseling-related field	Examination; supervisory agreement required	Counseling under supervision; client severity restricted	No

Abbreviations: Authorized Services typology, assessment, screening, intake, or evaluation; BH, behavioral health; CM, case management, referral, or care coordination; Counseling, counseling or therapeutic intervention; Crisis services, crisis intervention or de-escalation; Hrs, hours; MH, mental health; N/A, not applicable; NS, not specified; PS, psychosocial skills training or behavioral modification; SO, function is under supervision only, no independent practice; TP, treatment planning; Yrs, years.

Of the 20 credentials identified, all specified a degree type for entry. The most common bachelor's degree requirements were general areas of study in mental health, behavioral health, or human services disciplines (*n* = 7), followed by a related or relevant field (*n* = 6); a nonspecified bachelor's degree (*n* = 4); a behavioral science degree (*n* = 1); a counseling-related field (*n* = 1); and enrollment in or completion of a bachelor's program (*n* = 1). The required number of education and experience hours varied substantially across states, ranging from 60 hours of didactic education to 10 000 hours of direct experience. The vast majority of identified credentials (90%; *n* = 18) specified a practice hour requirement, while only 2 did not. Lastly, 9 credentials offered multiple pathways for entering the role.

The most commonly authorized functions were case management, referral, and care coordination (*n* = 11), followed by psychosocial skills training or behavioral modification (*n* = 8), assessment, screening, or intake (*n* = 8), and treatment planning (*n* = 5). Nearly half of the roles (*n* = 9) included a description specifying that authorized functions were restricted to practice under supervision only. Notably, 5 roles had no specified scope of practice or authorized services, leaving the scope of permissible practice undefined and likely dependent on supervisor responsibilities.

### Bachelor's-level roles defined by administrative code or other state policy

Twenty-two states had bachelor's-level roles defined by administrative code or other state policy guidance, yielding 30 different titles (see Table 2). Six states (AZ, DE, OR, PA, SC, and WI) offered multiple role types defined by administrative code or other state policy. Assessment of the 30 identified roles revealed the most common regulatory mechanism to be administrative code (*n* = 20), followed by state agency guidance (*n* = 6), and Medicaid provider manuals (*n* = 4). The most common degree requirement was a bachelor's degree in a general area of mental health, behavioral health, or human services discipline (*n* = 17), followed by a nonspecified bachelor's degree (*n* = 7), social work or behavioral science (*n* = 3), psychology or mental health field (*n* = 1), behavior analysis (*n* = 1), and nursing or occupational therapy (*n* = 1). Practice requirements were specified for 14 of the 30 roles, ranging from 45 hours to 4 years of relevant experience, while the remaining 16 roles did not specify a number of required practice hours. Twelve of the 30 roles allow alternative entry pathways into the role.

**Table 2.** Bachelor's-level behavioral health occupations defined through state regulatory mechanisms, by state (*n* = 22).

State	Title	Regulatory mechanism	Degree required	Practice hours	Authorized services	Alternative entry pathway
AL	Qualified Mental Health Provider—Bachelor's	Administrative code	Mental health or human services field	N/A	Medication monitoring; treatment plan review; therapeutic monitoring	No
AZ	Behavioral Health Technician	Administrative code	BH or human services	1 yr full-time BH experience	Institution-defined (SO)	Yes
AZ	Behavioral Health Paraprofessional	Administrative code	BH or human services	N/A	Institution-defined (SO)	Yes
AR	Qualified Behavioral Health Professional (QBHP)	Administrative code	Nonspecified bachelor's	N/A	NS	Yes
CA	Mental Health Rehabilitation Specialist (MHRS)	Administrative code	Nonspecified bachelor's	4 yrs in MH setting	Assessment; counseling; CM; crisis services; PS (SO)	Yes
CT	Psychology Technician	Administrative code	Psychology or MH field	80 hrs	Psychological/neuropsychological testing, scoring, and documentation (SO)	No
DE	Certified Mental Health Screener	Administrative code	BH or human services	4 yrs direct clinical experience	Emergency screening; evaluation for involuntary treatment (SO)	Yes
DE	Bachelor's Level Mental Health Worker	State agency guidance	Social work or behavioral science	N/A	NS	No
DE	Paraprofessional Mental Health Worker	State agency guidance	Social work or behavioral science	N/A	NS	Yes
HI	Mental Health Specialist	State agency guidance	BH or human services	2 yrs providing clinical MH services	Assessment; diagnosis; counseling; crisis services; advocacy; consultation	No
ID	Bachelor's-Level Paraprofessional Behavioral Health Worker	Administrative code	Nonspecified bachelor's	N/A	CM; crisis services; skills training; ACT; partial hospitalization; SUD outpatient services	No
IL	Mental Health Professional	Administrative code	Nonspecified bachelor's	N/A	NS	Yes
MI	Child Mental Health Professional (CMHP)	Administrative code	BH or human services	3 yrs supervised experience	Counseling; PS; skills training for children and families	No
MN	Mental Health Behavioral Aide 2	Administrative code	Nonspecified bachelor's	N/A	PS (SO)	Yes
NM	Community Support Worker	Administrative code	BH or human services	1 yr relevant experience	CM; skills development; independent living support	No
NC	Qualified Mental Health Professional	Administrative code	BH or human services	2 yrs full-time pre- or post-bachelor's MH experience	NS	Yes
NV	Qualified Mental Health Associate (QMHA)	Medicaid provider manual	BH or human services	4 yrs relevant experience	Assessment; crisis services; PS; CM; partial hospitalization; psychosocial rehabilitation	Yes
OK	Psychological Technician	Administrative code	BH or human services	N/A	Psychological and neuropsychological testing (SO)	No
OR	Behavioral Health Specialist 1	State agency guidance	BH or human services	N/A	Assessment; counseling; PS; TP; skills training	No
OR	Behavioral Health Specialist 2	State agency guidance	Nursing or occupational therapy	N/A	Assessment; counseling; TP; diagnosis; supervision of lower-level staff	No
OR	Child Behavioral Health Specialist	Administrative code	BH or human services	700 hrs	Prevention; PS; crisis services; evidence-based interventions for children and adolescents	No
PA	Behavioral Health Technician	Administrative code	Nonspecified bachelor's	N/A	PS; behavioral intervention for children and youth (SO)	Yes
PA	Mental Health Worker	Administrative code	Social work, psychology, nursing, rehabilitation, or activity therapies	N/A	Permitted services under supervision (SO)	Yes
SC	Human Service Professional	Medicaid provider manual	BH or human services	45 hrs	PS; crisis services; assessment; CM; SUD counseling; skills training	No
SC	Non-Mental Health Professional	Medicaid provider manual	Nonspecified bachelor's	3 yrs direct care with serious mental illness	Community MH services per individual service standards (SO)	No
TX	Qualified Mental Health Professional—Community Services (QMHP-CS)	Administrative code	BH or human services	N/A	Assessment (pre-admission screening); TP	No
WI	Other Qualified Mental Health Professional	Administrative code	BH or human services	1 yr providing MH services	NS	No
WI	Behavioral Treatment Therapist	Medicaid provider manual	Behavior analysis or related field	N/A	Comprehensive ASD treatment; behavioral intervention; caregiver training	No
WV	Service Provider, Bachelor's Level	State agency guidance	Human services	N/A	Counseling (SO); CM; therapeutic behavioral services	No
WY	Certified Mental Health Worker	Administrative code	Human services	100 direct contact hrs; 400 indirect hrs	Assessment; TP; prevention; supervised clinical practice	No

Abbreviations: Authorized Services typology, assessment, screening, intake, or evaluation; BH, behavioral health; CM, case management, referral, or care coordination; Counseling, counseling or therapeutic intervention; Crisis services, crisis intervention or de-escalation; Hrs, hours; MH, mental health; N/A, not applicable; NS, not specified; PS, psychosocial skills training or behavioral modification; SO, function is under supervision only, no independent practice; TP, treatment planning; Yrs, years.

## Discussion and policy implications

States that are expanding behavioral health service delivery require a well-trained pipeline of qualified workers who can meet increasing demand. Bachelor's-level and other non-graduate-degree behavioral health professionals represent a critical segment of the behavioral health workforce because they often deliver front-line services, support care coordination, and fill gaps in underserved areas that can help to free up licensed professionals to focus on clinical needs.^8^ The significant shortage of mental health providers, combined with increased demand for and limited access to care, has generated calls for better preparation and expansion of this segment of the workforce. However, findings of this study suggest considerable differences exist in titles, requirements, and scope of practice/authorized services for bachelor's-level behavioral health support roles across the US.

Overall, 29 states were identified as having at least one bachelor's-level behavioral health support role credential, with 50 different roles meeting criteria for inclusion in the policy analysis. Most states (66%; *n* = 19) have a single bachelor's-level role, while 10 states (34%) define 2 or more bachelor's-level roles with variability in degree requirements, practice hours, and scope of practice authorizing tasks associated with each role (e.g., AK, VA). Relatedly, scope of practice and allowable service delivery varied substantially, with some states defining specific tasks and functions (e.g., AL, ID, and NM) while others provided no identified scope of practice specifications or defined practice boundaries (e.g., NC, WI). These differences were also noted within the same state. For example, in Delaware, a *Certified Mental Health Screener*, a *Bachelor's-Level Mental Health Worker*, or *Paraprofessional Mental Health Worker* are 3 different titles with both defined and nonspecified scope of practice definitions (see Table 2). In Alaska, the state with the most bachelor's-level titles (*n* = 8), titles are credentialed by 2 different bodies, and degree requirements, practice hours, and scope of practice allowances also differ. Findings related to degree requirements reveal considerable heterogeneity in the educational pathways through which individuals may enter the bachelor's-level behavioral health workforce. Across most states, required degrees are related or adjacent to the field (e.g., psychology, social work, human services, or behavioral sciences). Notably, 4 states impose no behavioral health-specific degree requirement, requiring neither formal coursework nor requisite practice hours in the field for credentialing (AR, ID, IL, and MN). As a result, entrants into the behavioral health workforce in these states could range from individuals with directly relevant training to those whose undergraduate preparation is entirely unrelated to behavioral health.

Despite our efforts to characterize each state's bachelor's-level behavioral health support workforce and further understand pathways and credentialing for this growing segment of the behavioral health workforce, it remains unknown how these regulations ensure quality service delivery. Given the wide variability in education requirements, scope of practice definitions, and credentialing standards across states, and given that many of these roles are still new, it remains difficult to evaluate the preparation, appropriateness, and effectiveness of these workers. Because these roles lack consistency both within and across states, the following recommendations outline areas of potential commonality.

### Finding commonality across bachelor's-level workforce regulations

While complete uniformity in role titles and definitions may not be achievable across all US states given differing state-level authority over credentialing and scope of practice, policymakers should consider strategies to establish shared core competencies for training, clearly articulated education pathways to increase supply, and credentials with transferability across jurisdictions. The following discussion outlines these domains and offers innovative models that show promise for replication and lessons learned as other states expand this workforce.

#### Shared competencies

Establishing professional standards of care is key for ensuring an educated, trained, and capable workforce.^18^ Precedent for this approach exists in adjacent workforce areas, including core competency frameworks developed for community health workers,^19,20^ certified peer support specialists,^21^ and addiction counselors,^22^ each of which demonstrated that common standards can guide service delivery, inform training programs, shape job descriptions, and support both entry into the field and ongoing professional development. Competency development, in tandem with greater definition of emerging bachelor's-level support professions, could present an avenue for beginning the process of clarifying the roles this workforce serves. Given the variability in what tasks and functions states allow bachelor's-level support roles to perform, and the inconsistency in how this information was presented across states (e.g., by task, by role, or by supervisory oversight), findings highlight that many states have not yet clearly delineated the scope of practice for bachelor's-level behavioral health workers, even as they credential these individuals for workforce entry. These findings suggest an opportunity for states to learn from one another in defining consistent, competency-based scopes of practice for bachelor's-level support roles, which could in turn support workforce planning and inform Medicaid and licensure policy across the behavioral health system.

#### Leveraging established educational pathway models

Given substantial variation identified across bachelor's-level behavioral health support role credentials, existing professional pathways may provide useful models for strengthening consistency in education and training. While states allowing a general behavioral health bachelor's degree to meet requirements for a bachelor's-level support credential create a broad group to recruit from, there are limited opportunities for students to understand what roles or jobs their degree could fill. The established BSW licensure pathway offers a useful model, demonstrating how nationally recognized accreditation standards, competency-based education, and state licensure requirements can align^23,24^ to promote standardized preparation and professional role clarity for the bachelor's-level workforce.^25^ As newer bachelor's-level behavioral health support credentials continue to evolve, policymakers and professional organizations might consider whether elements of the BSW model could inform more consistent educational, training, and regulatory frameworks while preserving the flexibility needed to meet diverse workforce needs.

#### Transferability and mobility of bachelor's-level workforce

Although progress toward interstate mobility has been made for licensed behavioral health clinicians through the Counseling Compact, the Psychology Interjurisdictional Compact (PSYPACT), and the Social Work Licensure Compact,^26^ no analogous compacts currently exist for the bachelor's-level behavioral health workforce. The absence of consistent role definitions, titles, and credentialing requirements across states means the infrastructure needed to support interstate mobility for these workers is far from realized. As bachelor's-level roles become more formally defined and widely adopted, and as states scale behavioral health delivery through models such as Certified Community Behavioral Health Clinics and crisis response systems, developing pathways toward credential portability and cross-state recognition will be essential for ensuring that workforce investments translate into durable improvements in access to care.^27^

#### Education pathway guidance

A significant gap in the current landscape is the absence of clearly articulated education and training roadmaps for individuals pursuing bachelor's-level behavioral health careers. In many health professions, well-defined occupational pathways serve a dual function: they signal to prospective workers what preparation is required to enter a given role and help current workers understand how to advance within their field.^28^ Without accessible, published resources specifying the education, supervised experience, and training expectations associated with bachelor's-level behavioral health positions, awareness of post-graduation options within this workforce segment is likely to remain limited, constraining the growth of the pipeline before individuals even enter the workforce. Research across health professions more broadly suggests that early and sustained exposure to health care career pathways is essential to shaping occupational choice, as many students have already narrowed their career trajectories by the time they enter postsecondary education. Introducing behavioral health career options earlier in secondary and undergraduate education, such as through mentorship, experiential learning, and explicit occupational guidance, represents a concrete strategy for cultivating awareness of bachelor's-level roles and the steps required to fill them.^28^ Importantly, greater articulation of these pathways would also yield downstream regulatory and systems-level benefits, as clarity in how roles are defined and trained helps inform scope of practice determinations, payment policy, and the broader alignment of workforce supply with population need.^4^

### Innovative strategies for workforce expansion

A number of states have pursued novel approaches to recruiting and preparing individuals for bachelor's-level behavioral health roles, offering promising models for broader replication. In Washington State, registered apprenticeship pathways were developed through the Health Care Apprenticeship Consortium in partnership with the SEIU Healthcare 1199 NW Multi-Employer Training Fund, establishing structured earn-while-you-learn entry points into behavioral health technician, peer counselor, and substance use disorder professional roles.^29^ Concurrently, the University of Washington collaborated with state partners to design a bachelor's-level Behavioral Health Support Specialist curriculum tied directly to a formal credential, with the explicit aim of expanding access to evidence-based psychosocial interventions in primary care and other underserved settings.^8,10^ In Utah, Senate Bill 26, signed into law in March 2024, sought to remove barriers to licensure and expand the behavioral health workforce by creating new entry-level certifications and establishing a pathway for individuals with bachelor's degrees to become licensed as Behavioral Health Coaches.^30^ The legislation responded directly to state data showing that roughly three-quarters of Utah's behavioral health workforce held a master's degree or higher, leaving a significant gap in the career ladder for bachelor's-level workers seeking to enter the field.^11^ In Massachusetts, William James College piloted the Behavioral Health Service Corps (BHSC), a year-long, paid service-learning program designed to recruit recent bachelor's degree graduates into community-based behavioral health organizations, pairing direct practice experience with academic coursework and mentorship to support both workforce entry and longer-term career development in the field.^12,31^ Taken together, these efforts reflect a broader state-level trend toward fostering partnerships with academic institutions to build career pipelines and develop new bachelor's-level providers as a strategy to expand behavioral health workforce capacity.

Likewise, for states that have not yet developed clear articulation of the bachelor's-level workforce, efforts to develop and define these credentials for existing, non-credentialed bachelor's-level positions could increase visibility and support of this workforce. Credentialing verifies that an individual has met qualifications to hold a particular position, including knowledge, skills, and training. Because credentialing ensures compliance with state and federal regulations, it is essential to maintaining ethical and professional standards of care. Formal credentialing may also enhance the trustworthiness of behavioral health practices to both clients and insurers and increase practice credibility within the community. Lastly, credential expansion could help inform billing eligibility and reimbursement for services provided by bachelor's-level providers.^32^

### Limitations

The search strategy relied on publicly available credentialing authority websites, administrative code, and state websites, and may not have captured all existing credentials, particularly those established through less visible regulatory mechanisms or recently amended statutes. Findings reflect the state of credentialing policy as of May 2025 and do not capture pending legislation or regulatory changes enacted after that date, as this is an emerging area of policy that states are actively expanding credentialing mechanisms. This analysis focuses on formal credential requirements as written in policy and regulatory documents, which may not fully reflect how bachelor's-level roles are implemented or deployed in practice across states. Lastly, our findings do not represent all bachelor's-level behavioral health workforce pathways (e.g., BSW and addiction counselors), and findings will continue to evolve as new or emerging bachelor's-level roles are introduced in the future.

## Conclusion

Findings underscore the significant variability in how bachelor's-level behavioral health support roles are currently defined and regulated across states. Understanding the current landscape is a prerequisite to building the systems needed to train and deploy bachelor's-level workers in response to growing behavioral health demand. The policy strategies best suited to address this vary by credentialing mechanism. For states with formal licensure or certification structures, priorities may include expanding scope of practice, aligning educational requirements with workforce need, and improving credential portability across jurisdictions. For roles defined through administrative code, opportunities exist to formalize oversight, clarify training expectations, and establish pathways for advancement. For states where bachelor's-level support roles are defined only through agency guidance or Medicaid policy, the most immediate lever may be elevating these roles into other regulatory frameworks that can support consistent recruitment, training, and reimbursement. Across all mechanisms, increasing transparency in how roles are defined and what pathways exist to fill them is a shared foundation for any state seeking to grow this workforce.

## Supplementary Material

qxag222_Supplementary_Data
