# Supplementary material for: Characterizing the credentialed bachelor's-level behavioral health support workforce: a national descriptive study
Source: Health Aff Sch. 2026 Sep 11;4(9):qxag222. doi: 10.1093/haschl/qxag222 (PMC13655456; doi:10.1093/haschl/qxag222)
Supplement: Supplementary file 1 [file qxag222_Supplementary_Data.zip › Appendix A.docx]

Appendix A. Extraction Guide

| **Variable Name** | **Definition** | **Coding** |
| --- | --- | --- |
| State | State in which the credential is established. | Open Text (state postal abbreviation). |
| Title of Role | Formal title of the credential, as named in the governing statute, administrative code, licensing board documentation, or agency policy. | Open Text. |
| Link to Documentation | Citation or hyperlink to the primary source document used to identify and verify the credential, such as a state administrative code, credentialing authority or licensing board website, Medicaid provider manual, or other government webpage. | Open Text. |
| **Credentialing and Regulatory Framework** | | |
| Licensure | Administered through a state occupational or agency licensing board; typically defines a specific scope of practice delineating authorized and unauthorized services. | 0 = No, 1 = Yes. |
| Certification | Offered through a state credentialing authority or an affiliate of a national credentialing organization; verifies that an individual has met defined educational and training standards but does not always carry a formally defined scope of practice. | 0 = No, 1 = Yes. |
| Administrative Code | A state legislature or agency establishes definitions, titles, and requirements for the role through rulemaking, without a corresponding licensure or certification structure. | 0 = No, 1 = Yes. |
| Medicaid Provider Manual | A state Medicaid agency or its contracted partners define eligible provider types and staffing qualifications outside of the formal rulemaking process. | 0 = No, 1 = Yes. |
| State Agency Guidance | State agency operational standards or program documents define the role outside of formal rulemaking. | 0 = No, 1 = Yes. |
| Other Documented State Policy | Any additional documented mechanism through which a state defines the role; please specify the policy mechanism if applicable. | 0 = No, 1 = Yes. |
| **Regulatory Authority Type** | | |
| Internal | Oversight body is a state government agency (e.g., a state department of health or department of human services). | 0 = No, 1 = Yes. |
| External | Credentialing body is an independent organization operating outside of state government (e.g., a professional certification board, a state affiliate of a national credentialing organization, or a professional association). | 0 = No, 1 = Yes. |
| **Educational Requirements (Type of Bachelor’s Degree; Select all that apply)** | | |
| Mental Health | Degree field required for the credential. | 0 = No, 1 = Yes. |
| Behavioral Health | Degree field required for the credential. | 0 = No, 1 = Yes. |
| Human Services | Degree field required for the credential. | 0 = No, 1 = Yes. |
| Behavioral Science | Degree field required for the credential. | 0 = No, 1 = Yes. |
| Social Work | Degree field required for the credential. | 0 = No, 1 = Yes. |
| Psychology | Degree field required for the credential. | 0 = No, 1 = Yes. |
| Counseling-Related Field | Degree field required for the credential. | 0 = No, 1 = Yes. |
| Related or Relevant Field | Degree field required for the credential. | 0 = No, 1 = Yes. |
| Other Bachelor's Degree | A specified bachelor's degree field not captured by the categories above; please specify the degree type if applicable. | 0 = No, 1 = Yes.; Open Text |
| No Major Specified | No specific degree field was required by the state. | 0 = No, 1 = Yes. |
| **Supervised Practice Requirements** | | |
| Supervised Hours Specified | State specifies a required number of supervised practice hours; please specify the number of hours if applicable. | Open Text; N/A if Not Applicable |
| Years of Experience Specified | State specifies a required number of years of experience; please specify the number of years if applicable. | Open Text; N/A if Not Applicable |
| Direct Client Contact Hours Specified | State specifies a required number of direct client contact hours; please specify the number of hours if applicable. | Open Text; N/A if Not Applicable |
| Other Experiential Requirement | Any other experiential requirement described by the state (e.g., didactic education hours); please specify if applicable. | Open Text; N/A if Not Applicable |
| Not Specified | No supervised practice requirement was described in the source documentation. | 0 = No, 1 = Yes. |
| **Alternative Pathway to Credential** | | |
| Alternative Pathway Available | State allows the standard bachelor's-level degree requirement to be substituted with specified work experience or supervised practice requirements. | 0 = No, 1 = Yes. |
| Alternative Pathway Degree | Substitute minimum degree or education level permitted (e.g., an associate's degree or high school diploma) in place of the standard bachelor's-level requirement. | Open Text |
| Alternative Pathway Supervised Practice Requirements | Additional supervised hours, years of experience, or other experiential requirements required to qualify through the alternative pathway. | Open Text |
| **Scope of Practice / Authorized Services** | | |
| Described authorized services and permissible functions using different terminology and varying levels of detail across statutes, administrative codes, and agency documentation. To allow for comparison across states, authorized services were grouped into the categories listed below; a credential could be assigned to more than one category. For each category, coders reviewed the source documentation to determine whether the service was authorized, and if so, whether the state described that service as requiring the oversight of a licensed or otherwise qualified supervisor. Categories where authorization was granted but restricted to supervised performance were coded as Supervision Only (SO). If no authorized services were not described at all in the source documentation, the credential was coded as Not Specified. If specific authorized services were not included in documentation it was coded as 0=No. | | |
| Assessment | Screening, intake, or assessment. | 0 = No; 1 = Yes; 2 = Yes, with Supervision Only (SO). |
| Treatment Planning | Development of an individualized treatment plan. | 0 = No; 1 = Yes; 2 = Yes, with Supervision Only (SO). |
| Counseling or Therapeutic Intervention | Provision of counseling or another therapeutic intervention. | 0 = No; 1 = Yes; 2 = Yes, with Supervision Only (SO). |
| Case Management, Referral, or Care Coordination | Case management, referral, or coordination of care. | 0 = No; 1 = Yes; 2 = Yes, with Supervision Only (SO). |
| Crisis Intervention or De-escalation | Crisis intervention or de-escalation services. | 0 = No; 1 = Yes; 2 = Yes, with Supervision Only (SO). |
| Psychosocial Skills Training or Behavioral Modification | Psychosocial skills training or behavioral modification services. | 0 = No; 1 = Yes; 2 = Yes, with Supervision Only (SO). |
| Prevention and Community Education | Prevention services or community education. | 0 = No; 1 = Yes; 2 = Yes, with Supervision Only (SO). |
| Clinical Supervision or Team Leadership | Supervision of lower-level staff or clinical team leadership. | 0 = No; 1 = Yes; 2 = Yes, with Supervision Only (SO). |
| Supervised Practice Only | No independent practice authority; function is under supervision only. | 0 = No, 1 = Yes. |
| Not Specified | Authorized services were not described in the source documentation. | 0 = No, 1 = Yes. |
